# Elastic properties of superconductors and materials with weakly correlated spins

**DOI:** 10.1038/s41598-017-05238-8

**Published:** 2017-07-07

**Authors:** Christian Binek

**Affiliations:** Department of Physics and Astronomy, University of Nebraska-Lincoln, Lincoln, NE 68588 USA

## Abstract

It is shown that in the ergodic regime, the temperature dependence of Young’s modulus is solely determined by the magnetic properties of a material. For the large class of materials with paramagnetic or diamagnetic response, simple functional forms of the temperature derivative of Young’s modulus are derived and compared with experimental data and empirical results. Superconducting materials in the Meissner phase are ideal diamagnets. As such, they display remarkable elastic properties. Constant diamagnetic susceptibility gives rise to a temperature independent elastic modulus for ceramic and single crystalline superconductors alike. The thermodynamic approach established in this report, paves the way to tailor elastic material parameters through the design of magnetic properties.

## Introduction

Some of the most powerful properties of equilibrium thermodynamics originate from the fact that the formalism implicitly includes interaction between various degrees of freedom such as the elastic, electric, and magnetic ones. Exchange of energy between different modes is built-in through the requirement of thermal equilibrium without reference to a specific microscopic mechanism. Because of this, thermodynamics establishes rigorous relations among equilibrium properties such as Maxwell and Clausius Clapeyron relations rather than model dependent approximations^1^.

A particular form of Maxwell’s relation is widely employed in the field of magnetocaloric materials^2^. Isothermal magnetic field-induced entropy change ${\rm{\Delta }}S$ of a material originates from magnetic and other degrees of freedom. Elastic degrees of freedom can significantly contribute to ${\rm{\Delta }}S$. Nevertheless, ${\rm{\Delta }}S$ is completely determined by the temperature and field dependence of the magnetization without reference to elastic properties^3, 4^.

This report utilizes the powerful property of equilibrium thermodynamics to relate magnetic and elastic properties. Simple, generic expressions for the relative temperature variation of Young’s modulus, *E*, are provided for temperature regimes of reversible equilibrium where materials show simultaneously linear magnetization response of paramagnetic or diamagnetic type and linear stress-strain relation. Results derived for the case of paramagnetic response can also be applied in the high temperature limit of ferroic materials. Superconductivity resembles an ideal realization of diamagnetism giving rise to the remarkably simple result of a temperature invariant elastic modulus in the Meissner phase. The possibility to express the temperature dependence of elastic parameters solely in terms of magnetic material properties paves the way to tailor elasticity by magnetic design.

A prominent example for the technological relevance of elastic properties of materials is the space shuttle Challenger accident where low ambient temperature at launch day caused hardening of rubber O-rings in a solid rocket booster with subsequent catastrophic failure^5^. In the scientific context, interest in the *T*-dependence of elastic properties has revived in a variety of fields. Those range from observations of abnormal elastic properties in Earth’s lower mantel^6^ to the investigation of magnetic field dependence of phonons in diamagnetic materials^7^. They further include magnetocaloric, elastocaloric, and barocaloric phenomena with modern cooling applications, and encompass new frontiers in the design of artificial materials^8–18^. Recently, microarchitectures with effective negative thermal expansion have been designed through structural integration of constituents with dissimilar but positive thermal expansion coefficients^19^. Combining the reported advances in microarchitecture with thermal properties of materials such as Invar and anti-Invar alloys may broaden the spectrum of applications^20^. Thermodynamics is an important tool in understanding the effective temperature response, properties of new materials for advanced cooling technologies, and fundamental relations between elasticity and magnetism.

Thermodynamic relations between magnetic and elastic properties are well-established in the literature. For example, in materials exhibiting the giant magnetocaloric effect, a first order magnetic phase transition accompanies a structural transition. The magneto-structural transition is an extreme manifestation of interaction between magnetic and elastic degrees of freedom^9, 21, 22^. Pasquale *et al*. evaluated ${\rm{\Delta }}S$ for the Heusler alloy Ni_55_Mn_20_Ga_25_ at the *H*-field induced first order transition^23^. At the transition, the alloy changes from a superelastic austenite to a pseudo-elastic martensite phase. A discontinuity, Δ*M*, in magnetization accompanies the lattice contraction. Pasquale *et al*. used the experimental Δ*M*-value and the magnetic Clausius-Clapeyron relation ${\rm{\Delta }}S=-{\mu }_{0}V{\rm{\Delta }}M\,\frac{{\rm{d}}H}{{\rm{d}}T}$ to calculate the isothermal entropy change^11, 23^. They found good agreement with the complementary evaluation of Δ*S* using the stress-strain data $\sigma ({\epsilon })$ of Ni_55_Mn_20_Ga_25_. With the help of ${\rm{\Delta }}S=-V{{\epsilon }}_{x}\,\frac{{\rm{d}}\sigma }{{\rm{d}}T}$ they numerically showed that Δ*S* can be determined from either the magnetic or the elastic Clausius Clapeyron equation. Their example implies ${\mu }_{0}{\rm{\Delta }}M\,\frac{{\rm{d}}H}{{\rm{d}}T}={{\epsilon }}_{x}\,\frac{{\rm{d}}\sigma }{{\rm{d}}T}$ and thus establishes a relation between magnetic and elastic properties. Here ${{\epsilon }}_{x}\approx 6 \%$ is the relative lattice contraction of Ni_55_Mn_20_Ga_25_.

In superelastic alloys such Ni-Ti doped with Cu, Co or Pd it is customary to calculate ${\rm{\Delta }}S$ from integration of the Maxwell relation ${(\frac{\partial s}{\partial \sigma })}_{T}={(\frac{\partial {\epsilon }}{\partial T})}_{\sigma }$ ^24^. Maxwell relations follow from the analytic behavior of thermodynamic potentials. Formally, the mixed partial derivatives of a thermodynamic potential depend on the sequence of differentiation at a first order phase transition. However, it has been shown that numerical integration of the Maxwell relation yields the correct entropy change when employing ${(\frac{\partial s}{\partial H})}_{T}={\mu }_{0}{(\frac{\partial M}{\partial T})}_{H}$ for magnetocaloric materials or ${(\frac{\partial s}{\partial \sigma })}_{T}={(\frac{\partial {\epsilon }}{\partial T})}_{\sigma }$ for elastocaloric materials close to the first order transition^25^. In addition to the indirect derivation of entropy change from magnetization data, *S*(*H*, *T*) can be determined from heat capacity measurements via $S(H,-T)={\int }_{0}^{T}\frac{C(H,T)}{T}dT$. Both methods have been successfully employed and critically compared^21, 25, 26^.

Atherton *et al*. showed the validity of ${(\frac{\partial {\epsilon }}{\partial H})}_{\sigma ,T}={\mu }_{0}{(\frac{\partial M}{\partial \sigma })}_{H,T}$, illustrating the correlation between magnetostrictive and magneto-mechanical effects^27^. Similarly, Bozoroth reports fair agreement between ${(\frac{\partial \epsilon }{\partial {\rm{H}}})}_{{\rm{\sigma }},{\rm{T}}}$ and ${{\rm{\mu }}}_{0}{(\frac{\partial {\rm{M}}}{\partial {\rm{\sigma }}})}_{{\rm{H}},{\rm{T}}}$ when evaluating experimental data of Permalloy^28^. Such investigations show that elastic properties are intimately related to magnetic properties of materials. However, none of the relations makes quantitative predictions about the *T*-dependence of an elastic constant such as Young’s modulus solely based on magnetic input without reference to stress, strain, or lattice properties.

Modeling of the temperature dependence of Young’s modulus is involved. In contrast to approaches based on microscopic models and statistical mechanics it is shown here that universal results can be derived for materials with generic magnetic properties. No reference to lattice properties of the material, the interatomic potential, defects, grain size, stress or strain is required. The rich experience in characterization and tailoring of magnetic properties can potentially be exploited to design elastic properties in temperature regimes with ergodic magnetic behavior.

## Thermodynamic derivation of the relative temperature change of Young’s modulus

As a starting point the differential, $dg$, of the Gibbs free energy density *g = G*/*V* is considered with *V* being the sample volume. Therefore, the validity of all subsequently derived relations is limited to the framework of reversible thermodynamics. To keep the notation simple and because experimental data of polycrystalline materials are considered, the elastic work differential is written in terms of scalar variables for stress, $\sigma$, and strain, ${\epsilon }$. The simplified differential form reads

1 $$dg=-sdT-{\mu }_{0}M\,dH-{\epsilon }\,d\sigma .$$

From the identity $\frac{{\partial }^{2}g}{\partial H\partial \sigma }=\frac{{\partial }^{2}g}{\partial \sigma \partial H}$ one obtains the established Maxwell relation ${(\frac{\partial {\epsilon }}{\partial H})}_{\sigma ,T}={\mu }_{0}{(\frac{\partial M}{\partial \sigma })}_{H,T}$, a thermodynamic relation for the Villari or inverse magnetostrictive effect, i.e. the change in magnetization due to applied stress^28^. From Legendre transformation of *g* one obtains $\tilde{g}=g-{\epsilon }\sigma$ and the differential form

2 $$d\tilde{g}=-sdT-{\mu }_{0}M\,dH+\sigma \,d{\epsilon }$$

with its three corresponding Maxwell relations

2a $${(\frac{\partial s}{\partial H})}_{{\epsilon },T}={\mu }_{0}{(\frac{\partial M}{\partial T})}_{{\epsilon },H}$$

2b $${(\frac{\partial s}{\partial {\epsilon }})}_{H,T}=-{(\frac{\partial \sigma }{\partial T})}_{{\epsilon },H}$$

2c $${(\frac{\partial \sigma }{\partial H})}_{{\epsilon },T}=-{\mu }_{0}{(\frac{\partial M}{\partial {\epsilon }})}_{H,T}$$

Integration of Eq. (2a) for the special case ${\epsilon }=0$ yields the well-known expression

3 $${\rm{\Delta }}{s}_{{H}_{f}}=s({H}_{f},T)-s(H=0,T)={\mu }_{0}{\int }_{0}^{{H}_{f}}{(\frac{\partial M}{\partial T})}_{{\epsilon }=0,H}dH$$

frequently applied to calculate the isothermal entropy change of magnetocaloric materials. Integration of Eq. (2b) for the special case *H* = 0 yields the isothermal entropy change

4 $${\rm{\Delta }}{s}_{{{\epsilon }}_{f}}=s({{\epsilon }}_{f},T)-s({\epsilon }=0,T)=-{\int }_{0}^{{\epsilon }{}_{f}}{(\frac{\partial \sigma }{\partial T})}_{{\epsilon },H=0}d{\epsilon }$$

applied in the case of elastocaloric materials. Using Eq. (4) with the general stress-strain relation in the Hooke limit $\sigma ={\epsilon }E$ yields

5 $${\rm{\Delta }}{s}_{{{\epsilon }}_{f}}=-\frac{1}{2}{\epsilon }{\,}_{f}^{2}{(\frac{\partial E}{\partial T})}_{H=0}.$$

In analogy to the approach by Pasquale *et al*. one expects a relation between ${\rm{\Delta }}{s}_{H}$ and ${\rm{\Delta }}{s}_{{\epsilon }}$. Specifically, one expects that the same magnitude of isothermal entropy change obtained from straining a material by a small amount ${{\epsilon }}_{f}$ in *H* = 0 can be induced under ${\epsilon }$ = 0 condition when applying a magnetic field *H*_*f*_ such that $|{\rm{\Delta }}{s}_{{H}_{f}}|=|{\rm{\Delta }}{s}_{{{\epsilon }}_{f}}|$. For materials with ${(\frac{\partial M}{\partial T})}_{{\epsilon }=0,H} < 0$, such as paramagnets, ${\rm{\Delta }}{s}_{{H}_{f}}$ is negative. For materials with ${(\frac{\partial M}{\partial T})}_{{\epsilon }=0,H} > 0$, such as metamagnets below the critical temperature, the entropy change is positive^29^. In paramagnets, ${\rm{\Delta }}{s}_{{H}_{f}} < 0$ originates from the fact that a magnetic field has the tendency to align magnetic moments through minimization of the Zeeman energy. Similarly, the ${\epsilon }$ -induced entropy change can display both a positive and a negative sign. Although ${\rm{\Delta }}{s}_{{\epsilon }} < 0$ is less common, it is observed for instance in rubber where stretching of the material reduces the number of possible configurations of the polymer molecules and thus reduces entropy^30^.

In the case of an elastic material with paramagnetic magnetization response

6 $$M({\epsilon }=0,H,T)=C\frac{H}{T},$$

and $C=const$, ${H}_{f}$ can be found by equating the reversible magnetic and elastic work according to

7 $$|{\mu }_{0}{\int }_{0}^{{{\rm{H}}}_{{\rm{f}}}}{\rm{M}}\,{\rm{d}}{\rm{H}}|={\int }_{0}^{{\epsilon }_{f}}\sigma \,d\epsilon .$$

The conditions under which Eq. (7) provides ${H}_{f}({{\epsilon }}_{f})$ such that $|{\rm{\Delta }}{s}_{{H}_{f}}|=|{\rm{\Delta }}{s}_{{{\epsilon }}_{f}}|\,\,$require some consideration. In fact, validity of Eq. (7) constraints the possible magnetic field responses to simple, ideal gas type, linear functional forms similar to the one given by Eq. (6). Next, general results of the formalism obtained for paramagnetic response are provided. Calculating ${\rm{\Delta }}{s}_{{H}_{f}}$ with the help of Eq. (6), utilizing the ${\rm{\Delta }}{s}_{{{\epsilon }}_{f}}$-expression provided by Eq. (5), and applying the entropy condition ${\rm{\Delta }}{s}_{{H}_{f}}=\pm {\rm{\Delta }}{s}_{{{\epsilon }}_{f}}$ together with the work condition of Eq. (7) yields

8 $${(\frac{\partial E}{\partial T})}_{H=0}=\pm \frac{E}{T}.$$

Eq. (8) can be compared with results from a model where *E*(*T*) is explicitly calculated from a given elastic equation of state $\sigma ({\epsilon },T)$. As a toy model, a material is considered which obeys the magnetic equation of state given by Eq. (6) and has the elastic properties of a rubber band which responds to tension according to

9 $$\sigma =T\frac{aL}{{L}_{0}}(1-{(\frac{{L}_{0}}{L})}^{3}),$$

where $a$ is a constant, ${L}_{0}$ is the *T*-independent length of the rubber band at $\sigma =0$, and $L$ the length of the rubber band for applied tension $\sigma$ ^31^. Linearization of the elastic equation of state (9) provides $E=3aT$ in agreement with the general result of Eq. (8) (see Supplemental Material for details).

**Figure 1:**
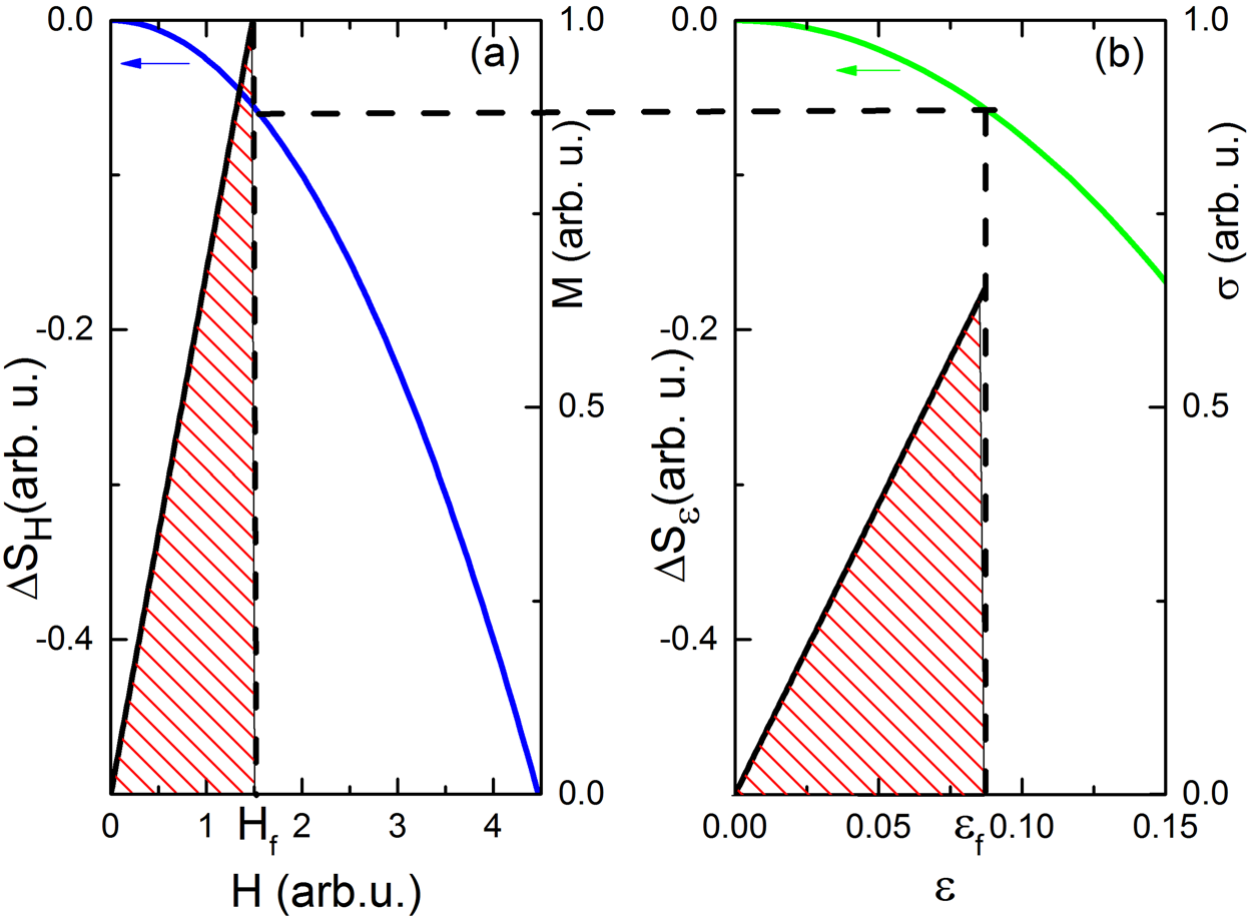
Thermodynamic Approach. (**a**) Blue curve depicts the isothermal entropy change ${\rm{\Delta }}{s}_{H}\,$(left axis). Black line shows the magnetization $M$ as a function of $H$ (right axis). The dashed area resembles the magnetic work when exposing the sample to the magnetic field ${H}_{f}$. (**b**) Green curve depicts the isothermal entropy change ${\rm{\Delta }}{s}_{{\epsilon }}$ (left axis). Black line shows stress $\sigma$ as a function of ${\epsilon }$ (right axis). The dashed area resembles the elastic work when straining the sample to ${{\epsilon }}_{f}$. The horizontal dashed line indicates equal values of ${\rm{\Delta }}{s}_{{H}_{f}}$ and ${\rm{\Delta }}{s}_{{{\epsilon }}_{f}}$.

Figure 1 illustrates the general concept of the thermodynamic approach. The blue and green lines (color online) associated with the left axes in Fig. 1a and b show ${\rm{\Delta }}s(H)$ and ${\rm{\Delta }}s({\epsilon })$. The horizontal dashed line elucidates that for a given value ${{\epsilon }}_{f}$ there is a field ${H}_{f}$ such that $|{\rm{\Delta }}{s}_{{H}_{f}}|=|{\rm{\Delta }}{s}_{{{\epsilon }}_{f}}|$. The work condition which relates ${{\epsilon }}_{f}$ and ${H}_{f}$ is obtained by equating the magnetic and the elastic reversible work. Dashed regions of identical area are enclosed by the linear functions $M(H)$ and $\sigma ({\epsilon })$. They depict the magnitude of the reversible magnetic (Fig. 1a) and elastic (Fig. 1b) work. The geometric construction of equal area relates ${{\epsilon }}_{f}$ and ${H}_{f}$ in accordance with Eq. (7) and establishes together with the entropy condition the temperature dependence of Young’s modulus. Eq. (7) provides ${H}_{f}({{\epsilon }}_{f})$ in the absence of spin-spin correlations (see Supplemental Material). A potential generalization to cases which include spin correlation requires a refinement of the work criterion given by Eq. (7).

An important application of the thermodynamic approach includes materials with a magnetic equation of state given by Eq. (6), linear stress-strain relation, and ${\rm{\Delta }}{s}_{{\epsilon }} > 0$. These conditions are fulfilled, e.g., for ferromagnetic metals significantly above ${T}_{C}$. Without any additional input application of

10 $${\rm{\Delta }}{s}_{{H}_{f}}=-{\rm{\Delta }}{s}_{{{\epsilon }}_{f}}$$

yields

11 $$-\frac{E}{T}={(\frac{\partial E}{\partial T})}_{H=0}$$

as a special case of Eq. (8). Equation (11) is expected to hold for all materials with ${\rm{\Delta }}{s}_{{{\epsilon }}_{f}} > 0$ in the Hooke limit and paramagnetic magnetization response. Prominent examples are 3d metals for $T\gg {T}_{C}$. Reciprocity suggests that elastic properties affect the magnetic properties of a material. Subsequent discussion of Eq. (11) shows that this is the case. In the limit $T\to 0$, Eq. (11) loses validity as a consequence of Nernst’s theorem, i.e., ${(\frac{\partial E}{\partial T})}_{H=0}\to 0$ for $T\to 0$. For $E=const\ne 0$, the low temperature limit of *E*/*T* diverges. Resolution of this inconsistency requires that the magnetic equation of state deviates from the paramagnetic form of Eq. (6) in the limit $T\to 0$. In fact, quantum ground states of solids deviate from paramagnetism. For example, simple crystalline solids tend to either order magnetically or condensate into a superconducting ground state. In both cases the magnetization response is no longer given by Eq. (6). The case of superconducting material in the Meissner phase is discussed below in detail.

Not all materials show paramagnetic response at high temperature. However, all materials have diamagnetic response either exclusively or in linear superposition with other susceptibility contributions. The diamagnetic susceptibility gives rise to linear response in magnetization on an applied magnetic field in a wide field range. Again, the linear response and absence of spin-spin correlation ensures applicability of the thermodynamic approach in conjunction with the reversible work condition of Eq. (7). Results for the case of diamagnetic response are applicable for virtually all non-magnetic elastic materials. A general relation which includes Eq. (11) as a special case and is also applicable in the case of diamagnetic susceptibilities is derived from $|{\rm{\Delta }}{s}_{{H}_{f}}|=|{\rm{\Delta }}{s}_{{\epsilon }_{f}}|$ and the use of Eq. (7) for $M=\chi (T)H$. It yields

12 $$\frac{1}{E}{(\frac{{\rm{\partial }}E}{{\rm{\partial }}T})}_{H=0}=\pm \frac{{\rm{\partial }}ln|\chi |}{{\rm{\partial }}T}.$$

## Temperature independence of Young’s modulus of superconductors in the Meissner state

The temperature dependence of the diamagnetic susceptibility of most materials is small and often experimentally difficult to separate from other contributions. In addition, theoretical understanding of diamagnetism in solids is incomplete. Superconducting materials in the Meissner state are a prominent exception. Here diamagnetism, with $\chi =-1$ in the ideal case or $-1\le \chi =const < 0$ in the presence of defects, is a consequence of magnetic flux expulsion. With $\chi =const$ follows $\frac{\partial ln|\chi |}{\partial T}=\frac{1}{\chi }\frac{\partial \chi }{\partial T}=0$ and Eq. (12) yields $E(T)=const$ for all temperatures sufficiently below the transition into the superconducting state. Obviously, $E(T)=const$ is consistent with Nernst’s heat theorem. Remarkably, to the best of the author’s knowledge, the simple but profound result $E(T < {T}_{c})=const$ has not been discussed in the literature.

**Figure 2:**
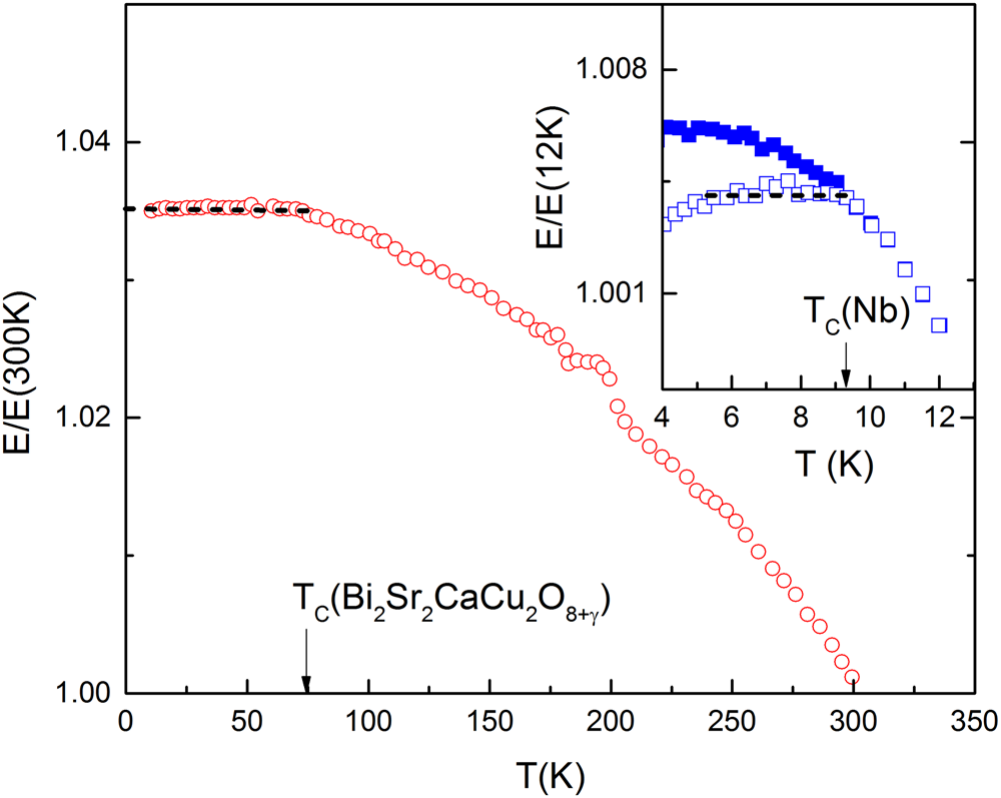
Elastic modulus of superconductors. Circles show $E/E(300K)$ vs $T$ of Bi_2_Sr_2_CaCu_2_O_8+γ_. The data are adapted from Lin *et al*.^32^. The predicted temperature invariance of the elastic modulus below ${T}_{C}\approx \,$8  K is indicated by a horizontal dashed line. The inset shows $E/E(12K)$ vs $T$ of Nb in zero magnetic field (open squares) and in the presence of an applied magnetic field (solid squares) $H > {H}_{c2}$ which destroys the superconducting state. Inset data are adapted from Pal-Val *et al*.^34^. The predicted temperature independence of the elastic modulus below ${T}_{C}\approx 9.3$ K is indicated by a horizontal dashed line.

Figure 2 shows the temperature dependence $E/E(T=300K)$ vs *T* (circles) of the cuprate superconducting ceramic Bi_2_Sr_2_CaCu_2_O_8+γ_ with a critical temperature of ${T}_{C}\,\approx \,$8  K. The data are adapted from Lin *et al*.^32^. The predicted temperature dependence $E(T < {T}_{C})=const$ (dashed line in Fig. 2) is virtually perfectly fulfilled in the entire temperature range $10\le T\le 80\,$K. Remarkably, $E(T < {T}_{C})=const$ as a special case of Eq. (12) holds despite the fact that cuprates are ceramic materials. Their ill-defined structural details are expected to affect the absolute value of $E$, but they do not affect the temperature dependence of the elastic modulus in the Meissner phase. Similarly, the measured compressional sound velocities in YBa_2_Cu_3_O_7−δ_ measured by Toulouse *et al*.^33^ clearly plateau for $T < {T}_{C}\,\approx \,$ 90K.

For the single crystalline BCS superconductor Nb, Eq. (12) is also applicable. The inset of Fig. 2 shows $E/E(T=12K)$ vs *T* of Nb measured under slow cooling condition in zero magnetic field (open squares) and in an applied magnetic field of *H* larger than the upper critical field *H*_*c*2_ = 376 kA/m (solid squares) (data are adapted from Pal-Val *et al*.^34^). Applicability of equilibrium thermodynamics requires a protocol of slow cooling. Therefore, only the data obtained on slow cooling and in zero field condition (inset Fig. 2 open squares) exhibit in good approximation plateau-like behavior for $T < {T}_{C}\,\approx \,$9.3 K as predicted by Eq. (12) (dashed line). The data taken in the presence of a strong magnetic field (inset Fig. 2 solid squares) continue to increase with decreasing temperature below ${T}_{C}$ because the Meissner phase is destroyed and the magnetic susceptibility is no longer constant.

## Comparison with experimental data and empirical *T*-dependencies of elastic constants

In order to obtain an explicit temperature dependence of Young’s modulus for diamagnetic materials other than superconductors, the temperature dependence of the diamagnetic susceptibility has to be known. Experimental and theoretical data concerning the temperature dependence of the diamagnetic susceptibility are scares. It is worth mentioning however, that the Langevin type contribution of valence electrons to the diamagnetic susceptibility, ${\chi }_{v}$, is related to the thermal expansion, $\alpha$. The *T*-dependence of ${\chi }_{v}\,\,$has been associated with $\alpha$ via the approximate functional form $\frac{dln{\chi }_{v}}{dT}=2\alpha$ ^35^. The *T*-dependence of lattice parameters resembles in good approximation the *T*-dependence of the internal energy, ${U}_{l}(T)$, of the lattice giving rise to $\alpha (T)=A\,{C}_{v}(T)$ with $A=const$ and ${C}_{v}=\partial {U}_{l}(T)/\partial T$ the heat capacity at constant volume. When substituting, $\frac{dln{\chi }_{v}}{dT}=2\alpha$, and $\alpha (T)=A\,{C}_{v}(T)$ into Eq. (12) one obtains

13 $$\frac{1}{E}{(\frac{\partial E}{\partial T})}_{H=0}=-2A{C}_{v}(T).$$

this expression holds for the large class of elastic materials with diamagnetic susceptibility. Note that the connection between $\frac{1}{E}{(\frac{\partial E}{\partial T})}_{H=0}$ and ${C}_{v}(T)$ has been made to compare Eq. (13) with results from the literature. It is important to stress that $\frac{1}{E}{(\frac{\partial E}{\partial T})}_{H=0}$ is determined by the diamagnetic susceptibility in accordance with Eq. (12).

Eq. (13) allows for comparison with empirical expressions for the *T*-dependence of elastic constants such as Wachtman’s equation $E(T)=E(T=0)-BT{e}^{-\frac{{T}_{0}}{T}}$ ^36^. Su *et al*. put Wachtman’s empirical formula on a microscopic basis^37^. Their work shows that ${T}_{0}$ can be identified with the Debye or Einstein temperature, ${\rm{\Theta }}$. For comparison between Wachtman’s expression and Eq. (13), ${C}_{v}(T)$ has to be considered in the Einstein approximation (see Supplemental Material). The Debye model provides a better approximation of ${C}_{v}(T)$ especially for the low temperature limit with ${C}_{v}(T)\propto {T}^{3}$. Substituting ${C}_{v}(T)\propto {T}^{3}$ into Eq. (13) implies $\frac{1}{E}{(\frac{\partial E}{\partial T})}_{H=0}\to -const\,{T}^{3}$ for $T\ll {T}_{0}$. In fact, the alternative empirical *T*-dependence $E(T)=E(T=0)[1-K\,{T}^{4}]$ with $K=const$ has been successfully applied to fit various experimental *E* vs *T* data^38^. It finds theoretical support by Born and Huang^39^. Limitations are discussed by Yarshni^40^. The asymptotic expression $\frac{1}{E}{(\frac{\partial E}{\partial T})}_{H=0}\to -\frac{4K}{{E}_{0}}{T}^{3}$ reflects Eq. (13) within the Debye approximation for $T\to 0$.

The above analysis shows that the *T*-dependence of Young’s modulus can be understood in terms of the magnetic field response of diamagnetic materials. The validity of the result is evidenced by comparison with empirical functional forms which have been successfully applied for data analysis in the literature. The empirical expressions have in part been motivated via thermodynamic and microscopic derivations which, however, refer to lattice properties of solids. The approach presented here relates *E*(*T*) to magnetic properties. They can be measured via magnetometry and potentially tuned via the vast toolbox available in the science of magnetic materials paving the way to magnetically tunable elastic properties. Next, Eq. (11), which has been derived for materials with paramagnetic response, is compared to data of Young’s modulus obtained from stress-strain relations in the literature.

**Figure 3:**
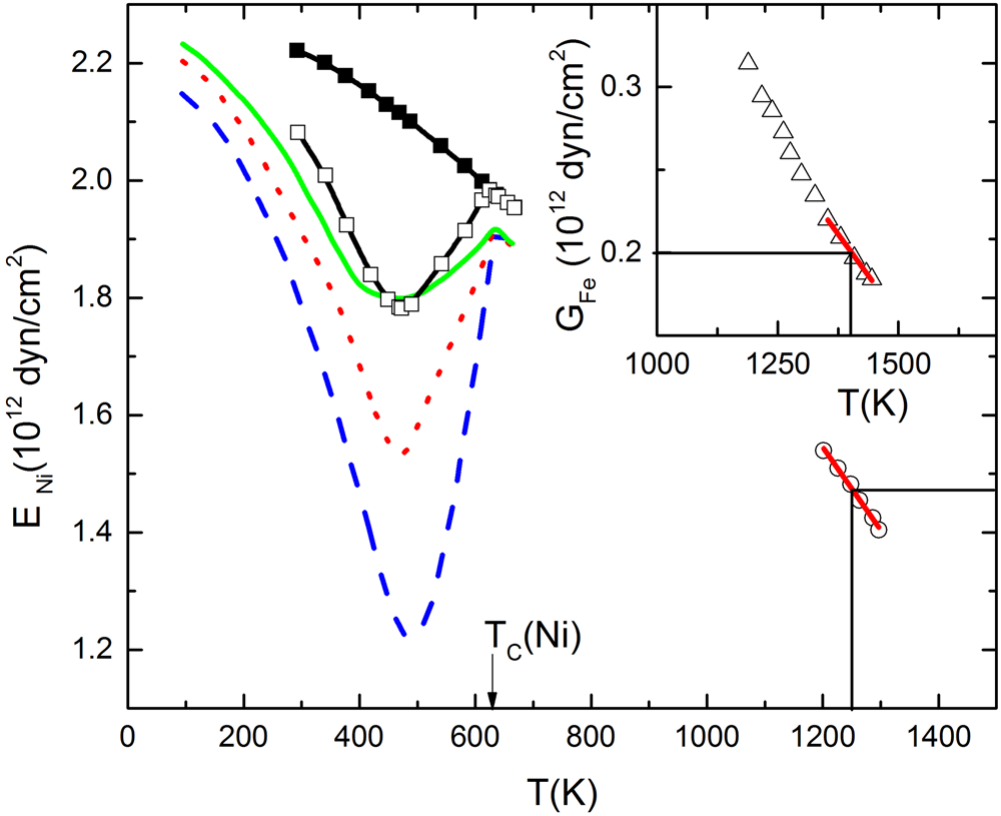
Elastic moduli of Ni and Fe. Dashed, dotted and solid lines show $E$ vs $T$ of Ni samples with grain sizes of 0.037, 0.0105 and 0.0095 mm. Squares show $E$ vs $T$ for a fourth sample in its demagnetized (open squares) and saturated state (solid squares). The arrow marks ${T}_{C}(Ni)$. Circles show $E$ vs $T$ of Ni in the high temperature regime. The line is a linear best fit to $E$ vs $T$ within $1200\le T\le 1300$ K. Inset shows the *T*-dependence of the shear modulus of Fe at temperatures above the bcc to fcc transition. The line is a linear best fit to $G$ vs $T$ within $1350\le T\le 1445$ K. All data are adapted from Ledbetter and Reed^38^.

Experimental data for Young’s modulus at high temperature and in particular data for 3d metals at temperatures substantially above ${T}_{C}$ are scarce. Perhaps the most comprehensive data collection is given in the review article by Ledbetter and Reed on elastic properties of metals and alloys, 1. Iron Nickel and Iron-Nickel alloys^38^. Figure 3 shows *E* vs *T* for Ni adapted from Ledbetter and Reed^38^. As expected, the magnetic state of a sample dramatically affects *E* at $T < {T}_{C}$. This is clearly seen when comparing data obtained with (Fig. 3 solid squares) and without (Fig. 3 open squares) applied magnetic field. Below ${T}_{C}$, the ${\rm{\Delta }}E$-effect is observed which quantifies the difference between *E* in a demagnetized and the magnetized state^41–44^. For $T\ge {T}_{C}$ there is virtually perfect collapse of $E(H,T)$ and $E(H=0,T)$ ^44, 45^. The Δ*E*-effect of polycrystalline Ni has been modelled by Hubert *et al*. using the linear temperature dependence of *E* vs. *T* in the reversible equilibrium regime at *T* > *T*_C_ as phenomenological input^46^. The entropy approach introduced in this manuscript is not applicable in the non-ergodic regime below ${T}_{C}$ and thus cannot describe the ${\rm{\Delta }}E$-effect. However, for $T > {T}_{C}$, i.e., in the absence of spontaneous magnetization when equilibrium thermodynamics is applicable, *E* becomes virtually independent from the applied magnetic field.

Similar to the disappearing of the ${\rm{\Delta }}E$-effect for $T > {T}_{C}$, differences in *E* between samples of different mechanical treatment become far less pronounced at $T > {T}_{C}$. Particularly conclusive is the perfect merging of *E* vs *T* at $T\ge {T}_{C}$ for the three cold worked samples shown in Fig. 3. The samples have been subject to different annealing protocols and show substantial variation in grain sizes ranging from 0.037 (Fig. 3 blue dashed line) to 0.0105 (red dotted line) and 0.0095 mm (green solid line). The data collapse for $T\ge {T}_{C}$ strongly supports the conclusion of a universal asymptotic high temperature limit of the relative temperature derivative of Young’s modulus. It is remarkable that in accordance with the thermodynamic prediction $\frac{1}{E}{(\frac{\partial E}{\partial T})}_{H=0}$ approaches a high temperature limit, which is independent from structural details as significant as grain sizes.

The temperature dependence of Young’s modulus of Ni is best suited for a quantitative comparison with Eq. (11). Open circles in Fig. 3 show *E* vs *T* data up to *T* = 1300 K taken by Armstrong and Brown^47^. A linear fit (line) of *E* vs *T* between $1200\le T\le 1300$ K provides ${(\frac{\partial E}{\partial T})}_{H=0,T=1250K}=-1.4\times {10}^{9}$ dyn/cm^2^K. The modulus at *T* = 1250 K, in the center of the fitting interval, reads *E*(*T*=1250 K) = $1.5\times {10}^{12}$ dyn/cm^2^ which yields $-\frac{E(T=1250K)}{1250K}=-1.2\times {10}^{9}$ dyn/cm^2^K. Comparison of the numerical values for ${(\frac{\partial E}{\partial T})}_{H=0,T=1250K}$ and $-\frac{E(T=1250K)}{1250K}$ validates Eq. (11) within an error of 14%. The residual discrepancy is expected when considering that the underlying assumption of correlation-free paramagnetic behavior is only approximately given at $T\approx 2{T}_{C}(Ni)$ with ${T}_{C}(Ni)=631\,K$.

The inset of Fig. 3 shows the *T*-dependence of the shear modulus, *G*, for fcc iron at temperatures $1200\le T\le 1450$ K (triangles). Data are adapted from Ledbetter and Reed^38^. Assuming that the Poisson ratio, $\nu$, is approximately constant in this temperature regime, $G\propto E$ is expected for polycrystalline Fe in accordance with $G=\frac{E}{2(1+\nu )}$ for isotropic materials. Within this approximation, Eq. (11) can be applied when substituting *E* by *G*. From a linear fit of *G* vs *T* at $1350\le T\le 1445\,$K (line in inset of Fig. 3) one obtains ${(\frac{\partial G}{\partial T})}_{H=0,T=1387.5K}=$ $-4.0\times {10}^{8}$ dyn/cm^2^K. With $G(T=1397.5K)=2.0\times {10}^{11}$ dyn/cm^2^ we obtain $-\frac{G(T=1397.5K)}{1387.5K}=-1.43\times {10}^{8}$ dyn/cm^2^K. Although $-\frac{G(T=1397.5K)}{1387.5K}$ and ${(\frac{\partial G}{\partial T})}_{H=0,T=1397.5K}$ are of the same order of magnitude, the discrepancy between the numerical values of ${(\frac{\partial G}{\partial T})}_{H=0}$ and $-G/T$ is much higher for Fe than in the case of Ni. This is expected due to the fact that ${T}_{C}(Fe)=1043K$ is significantly higher than ${T}_{C}(Ni)=631K$. Consequently, the deviation from uncorrelated paramagnetism is much more pronounced in the temperature regime available for Fe than it is for Ni.

## Conclusion

Thermodynamics relates the temperature dependence of the elastic modulus of a material solely to its magnetic properties. General results for the temperature derivative of Young’s modulus can be derived for paramagnetic materials, diamagnetic materials, and superconducting materials in the Meissner phase. The temperature independent diamagnetic susceptibility of a superconducting material below its critical temperature gives rise to the remarkably simple result that Young’s modulus is constant below the superconducting transition temperature for ceramic and single crystalline superconducting materials alike. For normal conducting diamagnetic materials, it is possible to find explicit functional forms for the temperature derivative of the elastic modulus. These findings and the generality of the formalism lead to the conclusion that, in the framework of reversible equilibrium thermodynamics, the temperature dependencies of elastic moduli are fully encoded in the magnetic properties of a material. Energy exchange between various degrees of freedom, which is implicitly taken into account in the framework of equilibrium thermodynamics, makes it possible that magnetic properties determine elastic moduli. This insight paves the way to tailor elastic properties by tuning magnetic properties beyond the known ${\rm{\Delta }}E$-effect. The results presented in this manuscript are obtained for simple magnetic equations of state where spin-spin correlation is absent. A refined approach, which includes spin correlation, could unfold its full potential for applications aiming at design of elastic properties. Future work of particular interest includes microscopic theoretical approaches, which explore quantitatively the absolute temperature dependencies of Young’s modulus for magnetic and superconducting materials.

## Electronic supplementary material

Supplementary Information
