# Supplementary material for: Elastic properties of superconductors and materials with weakly correlated spins
Source: Sci Rep. 2017 Jul 7;7:4906. doi: 10.1038/s41598-017-05238-8 (PMC5501875; doi:10.1038/s41598-017-05238-8)
Supplement: Supplementary file 1 — Supplementary Information [file 41598_2017_5238_MOESM1_ESM.pdf]

Supplementary Information

Elastic properties of superconductors and materials with weakly correlated spins

Christian Binek

Department of Physics and Astronomy, University of Nebraska-Lincoln, Lincoln, NE 68588

Temperature dependence of Young's modulus of a paramagnetic rubber band

With $\epsilon = \frac{L-L_0}{L_0}$, Eq.(9) takes the form

$$\sigma(\epsilon) = aT(1+\epsilon)\left(1 - \frac{1}{(1+\epsilon)^3}\right). \qquad \text{(S1)}$$

In the regime of small ϵ, Eq.(S1) simplifies into $\sigma(\epsilon) = \epsilon\, E$ where Young's modulus, E, is given by $E = 3aT$. The explicit T-dependence of Young's modulus is used below to validate the general thermodynamic methodology outlined in the manuscript. As a consistency check, Δs_{ϵ_f} can be calculated from Eq.(4) and Eq.(9) which yields $\Delta s_{\epsilon_f} = -\frac{a}{2}\epsilon_f^2 \frac{3+\epsilon_f}{1+\epsilon_f}$. Expansion of Δs_{ϵ_f} for small ϵ_f yields

$$\Delta s_{\epsilon_f} = -\frac{3a}{2}\epsilon_f^2. \qquad \text{(S2)}$$

Alternatively, Eq.(S2) is obtained when substituting the linearized expression $\left(\frac{\partial \sigma}{\partial T}\right)_{\epsilon,H=0} = \left(\frac{\partial E}{\partial T}\right)_{\epsilon,H=0} \epsilon = 3a\epsilon$ into Eq.(4). Using Eqs.(7) and (6) together with Hooke's linear stress-strain relation $\sigma(\epsilon) = \epsilon\, E$, i.e. without reference to a specific equation of state, one obtains

$$\mu_0 C \frac{H_f^2}{T} = \frac{1}{2}\epsilon_f^2 E. \qquad \text{(S3)}$$

Calculating Δs_{H_f} for $\left(\frac{\partial M}{\partial T}\right)_{\epsilon=0,H} = -C\frac{H}{T^2}$ yields $\Delta s_{H_f} = -\frac{1}{2}\mu_0 C \frac{H_f^2}{T^2}$. Using this result together with Eq.(S3) yields

$$\Delta s_{H_f} = -\frac{1}{2}\epsilon_f^2\frac{E}{T}. \qquad \text{(S4)}$$

Substitution of Eq.(S4) and Eq.(5) into $\Delta s_{H_f} = \Delta s_{\epsilon_f}$ yields $\frac{E}{T} = \left(\frac{\partial E}{\partial T}\right)_{H=0}$. In fact, for $E = 3aT$ one finds $\frac{E}{T} = 3a = \left(\frac{\partial E}{\partial T}\right)_{H=0}$.

Limitations of the formalism associated with spin-spin correlations

It is shown that in the presence of spin-spin correlations, Eq.(7) cannot be applied to find $H_f(\epsilon_f)$. For example, a ferromagnetic susceptibility of the classical Curie type $\chi \propto 1/(T - T_C)$ implies that in the limit $T \to T_C$ the work, $\int_0^{H_f} M\, dH$, increases to unbound values for any finite H_f. This unphysical behavior originates from the fact that $\chi \propto 1/(T - T_C)$ or more generally, $\chi \propto 1/(T - T_C)^\gamma$, are zero field susceptibilities defined according $\chi = \lim_{H\to 0}\left(\frac{\partial M}{\partial H}\right)_T$. The approximation $M = \chi(T)H$ neglects nonlinear terms which are crucial in the presence of spin-spin correlations. An attempt to find H_f from Eq.(7) when including the leading non-linear term via $M = \chi(T)H + \chi_{nl}(T)H^3$ fails as well. This becomes obvious when calculating $f := \Delta s_{H_f}/\mu_0 \int_0^{H_f} M\, dH$. For the nonlinear $M(H)$-expression f becomes H_f-dependent. However, consistency of the formalism requires $f = \mp\frac{1}{E}\left(\frac{\partial E}{\partial T}\right)_{H=0}$ independent of H_f or equivalently $\frac{\partial f}{\partial H_f} = 0$. A necessary condition for the validity of Eq.(7) as a tool to determine $H_f(\epsilon_f)$ is therefore the constraint $\frac{\partial f}{\partial H_f} = 0$. It restricts the functional forms of magnetic equations of state to linear response functions with negligible spin-spin correlation. Consequently, Eq.(7) cannot be applied near a critical point let alone below the critical temperature where long-range magnetic order sets in. Indeed, it is an experimentally established fact that for ferromagnetic alloys below the Curie temperature, Young's modulus depends very sensitively on the magnetic domain state of the material and is not a function of T alone. Equilibrium thermodynamics and the thus the formalism outlined in this manuscript is not applicable in the non-ergodic regime.

Comparison between Wachtman's equation and Eq.(13)

For comparison between Wachtman's equation and Eq.(13), $\frac{1}{E}\left(\frac{\partial E}{\partial T}\right)_{H=0}$ is calculated from $E(T) = E(T=0) - B\,T\,e^{-\frac{T_0}{T}}$ yielding

$$\frac{1}{E}\left(\frac{\partial E}{\partial T}\right)_{H=0} = \frac{\left(-Be^{-\frac{T_0}{T}}\left(1+\frac{T_0}{T}\right)\right)}{\left(E_0-B\,T\,e^{-\frac{T_0}{T}}\right)}. \qquad \text{(S5)}$$

Eq.(S5) and Eq.(13) share the limiting property $\frac{1}{E}\left(\frac{\partial E}{\partial T}\right)_{H=0} \to 0$ for $T \to 0$ in accordance with Nernst's heat theorem. It is interesting to note that, within the Einstein model for $C_v(T)$, Eq.(13) implies $\frac{1}{E}\left(\frac{\partial E}{\partial T}\right)_{H=0} \to \frac{const}{T^2} e^{-\frac{\hbar\omega_E}{k_B T}}$ for $T \ll \frac{\hbar\omega_E}{k_B} = T_0$ in close resemblance of the limiting expression $\frac{1}{E}\left(\frac{\partial E}{\partial T}\right)_{H=0} \to -\frac{BT_0}{E_0 T} e^{-\frac{T_0}{T}}$ obtained from Wachtman's equation.

The Einstein model is known to oversimplify the *T*-dependence of the heat capacity potentially explaining the difference in the *T*-dependent factors in front of the leading exponential terms.
